# Nutrition Prescription to Achieve Positive Outcomes in Chronic Kidney Disease: A Systematic Review

**DOI:** 10.3390/nu6010416

**Published:** 2014-01-22

**Authors:** Susan Ash, Katrina L. Campbell, Jessica Bogard, Anna Millichamp

**Affiliations:** 1 Institute of Health and Biomedical Innovation, School of Exercise and Nutrition Sciences, Queensland University of Technology, Brisbane 4059, Australia; 2 Princess Alexandra Hospital, Brisbane 4001, Australia; E-Mail: Katrina_cambpell@health.qld.gov.au; 3 School of Exercise and Nutrition Sciences, Queensland University of Technology, Brisbane 4059, Australia; E-Mails: Jessica.bogard@live.com (J.B.); a.spongberg@student.qut.edu.au (A.M.)

**Keywords:** chronic kidney disease, dietetics, evidence based practice, diet therapy, nutrition prescription

## Abstract

In Chronic Kidney Disease (CKD), management of diet is important in prevention of disease progression and symptom management, however evidence on nutrition prescription is limited. Recent international CKD guidelines and literature was reviewed to address the following question *“What is the appropriate nutrition prescription to achieve positive outcomes in adult patients with chronic kidney disease?”* Databases included in the search were Medline and CINAHL using EBSCOhost search engine, Embase and the Cochrane Database of Systematic Reviews published from 2000 to 2009. International guidelines pertaining to nutrition prescription in CKD were also reviewed from 2000 to 2013. Three hundred and eleven papers and eight guidelines were reviewed by three reviewers. Evidence was graded as per the National Health and Medical Research Council of Australia criteria. The evidence from thirty six papers was tabulated under the following headings: protein, weight loss, enteral support, vitamin D, sodium, fat, fibre, oral nutrition supplements, nutrition counselling, including protein and phosphate, nutrients in peritoneal dialysis solution and intradialytic parenteral nutrition, and was compared to international guidelines. While more evidence based studies are warranted, the customary nutrition prescription remains satisfactory with the exception of Vitamin D and phosphate. In these two areas, additional research is urgently needed given the potential of adverse outcomes for the CKD patient.

## 1. Introduction

Chronic kidney disease (CKD) is a prevalent chronic condition and the incidence of End-Stage Renal Disease (ESRD) is expected to continue to climb in the coming decade [1]. CKD has significant health and lifestyle implications for those affected, including increased risk of cardiovascular disease [2], malnutrition [3] and is a public health burden particularly in those patients who progress to end stage renal failure (or ESRD) and require kidney replacement therapy (dialysis) or transplantation [4]. The health cost burden is disproportionate to the prevalence with 5% of the health budget in the United States being consumed by 1% of the population requiring renal replacement [5]. CKD poses a significant public health issue and optimal treatment and management of this disease is indicated [6].

In CKD, nutrition and diet play an important role both in prevention of disease progression and in symptom management. The Dietitians Association Australia’s (DAA) *Evidence based guidelines for the nutritional management of Chronic Kidney Disease (CKD) stages 1–5* [7] provide statements of evidence against clinical questions in line with the Nutrition Care Process (NCP) [8]. The guidelines are designed to be employed by dietitians in clinical practice as the basis of nutritional management of patients with CKD and are based on the nutrition component of several recognized international guidelines. The evidence used, dates from published guidelines to 2005, and some of these guidelines varied in the method of rating evidence. Since 2006, a number of new international guidelines have been published or revised using an agreed grading system [9] and together with new literature these need to be reflected in dietetic practice, specifically the nutrition intervention or prescription employed by clinical dietitians. This article reviews the evidence presented in recent international guidelines and literature that address the clinical question *“What is the appropriate nutrition prescription to achieve positive outcomes in adult patients with chronic kidney disease?”*

## 2. Methods

A systematic literature review of studies was designed to answer the clinical question. Databases included in the search were Medline and CINAHL using EBSCOhost search engine, Embase and the Cochrane Database of Systematic Reviews. MeSH terms for Medline and CINAHL were “kidney failure, chronic” AND “diet therapy” OR “nutrition intervention” and for Cochrane “kidney failure, chronic”. M-tree headings in EMBASE were “chronic kidney disease” AND “diet therapy” and further derivatives of diet therapy such as protein, phosphate. Results were limited to those published from 2000 to 2009, papers reported in the English language and studies involving adult humans. Articles were excluded if they were not reported in full or if they were presented as tutorials, editorials, news, letters or comments. Articles were also excluded if they were included within any systematic reviews or meta-analyses retrieved. The research aims and outcome measures reported on were used to assess applicability of the studies. Reference lists of retrieved papers were also reviewed and studies included where relevant. Nutritional management of acute renal disease, transplantation and nephrotic syndrome were not included in this review.

In addition to this systematic literature search, hand searches of recognised international guidelines published since 2006 and pertaining to nutrition were conducted. These included:

- European Renal Association/European Dietitian and Transplant Nurses Association *ERA/EDTNA European Best Practice Guideline on Nutrition,* 2007 [10],
- *Guidelines for the management of chronic kidney disease* by the Canadian Medical Association, 2008 [11],
- *Diagnosis and management of chronic kidney disease: A national clinical guideline* by the Scottish Intercollegiate Guidelines Network, 2008 [12],
- National Institute for Health and Clinical Excellence in the United Kingdom *Chronic kidney disease: national clinical guideline for early identification and management in adults in primary and secondary care,* 2008 [13],
- Caring for Australians with Renal Impairment (also known as *CARI guidelines*), 2013 [14,15,16],
- Kidney Disease: Improving Global Outcomes (KDIGO): *Clinical practice guideline for the evaluation and management of Chronic Kidney Disease*, 2012 [5,17],
- American Dietetic Association *Chronic Kidney Disease Evidence-Based Nutrition Practice Guideline,* 2010 [18],
- British Dietetic Association *Evidence-based guidelines for the protein requirements of adults undergoing maintenance haemodialysis or peritoneal dialysis*, 2013 [19].

Papers obtained through the literature search were categorized according to the aspect of nutrition prescription addressed in the research question, for example protein, phosphate, fat, vitamin D, oral nutrition support. The strength of evidence of these papers was then assessed by three independent reviewers and categorized according to recommendations from the National Health and Medical Research Council (NHMRC) evidence hierarchy [20]. The NHMRC grades the level of evidence from I, a systematic review of all relevant randomized controlled trials to IV evidence obtained from case series. An overall grading of evidence is provided by NHMRC whereby level of evidence, consistency across studies, clinical impact and generalisability is also assessed from A, where the body of evidence can be trusted to guide practice to D were the body of evidence is weak and recommendations should be applied with caution. This system has been recognized as equivalent to the Kidney Disease Improving Global Outcomes (KDIGO) criteria [21].

In a similar fashion, evidence statements from international guidelines were then grouped according to aspects of the nutrition prescription addressed with their corresponding levels of evidence. The grading systems and definitions for levels of evidence and strength of practice recommendations used by the various institutions guidelines are compared in Appendix 1. Statements from the recent guidelines were then listed against each of the nutrition parameters defined above.

## 3. Results

Database searches using the search terms described above yielded 325 individual papers. Following grading of the evidence quality and exclusion of papers of lower level evidence, 34 papers remained for inclusion in this review. Of these, five papers were systematic reviews, fourteen were randomized controlled trials, nine were prospective cohort or comparative studies with controls and six were interrupted time series or case series. Table 1 compares the systematic reviews of protein intake both in those with and without diabetes, weight management and enteral support on renal outcomes. Table 2 compares the evidence for the remaining studies according to nutrient parameters, such as protein, vitamin D, fats, sodium, fibre; or intervention, such as oral nutrition support, dietetic counseling, including phosphate, nutrients in peritoneal solution, intradialytic parenteral nutrition or percutaneous endoscopic gastrostomy feeding. Table 3 outlines statements from international guidelines against each of these parameters.

In Table 1, the systematic reviews of protein intake [22,23,24] indicate that in pre-dialysis, protein should be reduced to 0.6 g/kg body weight/day or equivalent if using keto-analogues and diet combined. Reduction to this level resulted in a 32% reduction in renal deaths (RR 0.68, 95% CI 0.55 to 0.8, *p* *=* 0.0002). For those with diabetes, both Types 1 and 2, reducing protein is associated with moderate non-significant slowing in progression of diabetic nephropathy resulting in renal failure [24]. Protein intakes <0.8 g/kg body weight/day showed no compromise in anthropometry or biochemical indicators [23]. One systematic review was retrieved that examined weight loss interventions in CKD [25]. This review analysed 13 studies, two RCTs and 11 observational studies and found that only modest evidence exists to support the role of intentional weight loss on slowing CKD progression in mild-moderate CKD. A systematic review of enteral feeding in maintenance HD included 5 RCT and 13 non-RCT and concluded that enteral feeding, including oral nutrition support *vs*. routine care increased protein and energy intake and improved serum albumin by 0.23 g/dL but there was insufficient data to examine the effect on clinical outcomes [26].

Table 2 outlines individual papers. There is evidence that for those patients with ESRD either Stage 4 or 5, a very low protein diet (0.3 g/kg/day) with added keto-analogues and adequate energy (35 kcal/day) can delay dialysis with no adverse effect on mortality [27]. Elderly patients with glomerular filtration rate (GFR) between 5 and 7 mL/min on a similar diet, when compared to those on dialysis, had better outcomes with an improved survival of 3.6% (95% CI, −17 to +10; *p* *=* 0.002) [28]. In dialysis, protein intakes of >1.2 g/kg/day resulted in significant increases in body mass index (BMI, kg/m^2^) of 0.97 (*p* *<* 0.001) [29]. The association between protein intake and all-cause mortality and cardiovascular mortality amongst a large retrospective cohort, found that survival was best at protein intakes between 1.0 and 1.4 g/kg/day and that intakes <0.8 g/kg/day and >1.4 g/kg/day were associated with increased mortality. However, this effect was diminished significantly when adjusted for malnutrition inflammation complex syndrome [30].

**Table 1:** Systematic reviews of nutrition interventions in patients with chronic kidney disease (CKD).

Author	Number of Studies	Sample	Outcome Measures	Results	Conclusions	Level of Evidence [20]
**Protein (patients without diabetes)**						
Fouque [22]	10 RCT * s	*n* = 2000 Pre-dialysis, Stages 3–5	Renal death (death of any cause, requirement to start dialysis or kidney transplant)	RCTs or cross-over studies (if start date allocated randomly). Protein intake (≥0.8 g/kg/day) *vs.* moderate (0.6 g/kg/day) to severe protein restriction (0.3 g/kg/day) regardless of supplementation with amino acids or keto-acids. Participants with moderate to severe CKD * (as per GFR *, serum creatinine or creatinine clearance).	A nutritional intervention that includes a reduction in protein intake should be proposed to patients with moderate CRF *. Reducing protein intake overall reduced renal deaths by about 32% (*p* = 0.0002). Sub-analysis (7 studies) found that reduced protein intakes between 0.3 and 0.6 g/kg/day compared to higher/free protein intakes resulted in a significant reduction in renal deaths (37%, *p* = 0.0009).The optimal level of protein intake cannot be determined based on this review.	I
Zarazaga [23]	26 studies, Including 3 meta-analyses N.B. 3 studies included paediatric patients	*N* = 7155 Dialysis + Pre-dialysis (Stages not defined)	Compliance with diet Mortality, GFR, renal function Anthropometry Biochemistry (various factors that address overall renal function) Nutritional status	Patients aged 2–65 years with chronic renal failure in dialysis or pre-dialysis. Interventions of nutritional support with amino acid or keto-acid supplements with or without restriction of protein intake. Protein restricted to equivalent of 0.6 g/kg/day, energy 30–40 kcal/day and phosphate 700–800 mg/day in interventions.	Dietary protein should be restricted to 0.4–0.6 g/kg/day. A protein intake of 0.6 g/kg/day (comprising 0.4 g/kg/day + 0.2 g/kg/day from supplements) improves the course of renal function, nutritional status and lipid profile, with good compliance. VLPD * and LPD * (using specific enteral supplements) should be used by most patients in the early stages of CRF * to slow progression of renal failure. For patients with CRF on dialysis, prescription of a VLPD does not reduce frequency of dialysis sessions.	I
**Protein (patients with diabetes)**						
Robertson [24]	12 studies (9 RCTs and 3 before and after studies)	*n* = 585 (T1DM = 322, T2DM = 263)	Compliance with low protein diet Biochemistry (GFR) All- cause mortality, ESRD * Nutritional status, Health related QOL *, Costs	RCTs or before and after studies. Interventions of reduced or modified protein intake ≥4 months. Participants of any age with type 1 or 2 DM *, with nephropathy (UAER * ≥ 300 mg/day).	Reducing protein intake is associated with a moderate, non significant slowing in the progression of diabetic nephropathy to renal failure. A specific recommendation of the necessary protein level to achieve this outcome is not possible.	I
Zarazaga [23]	19 studies Including 1 systematic review	*N* = 280 Diabetic nephropathy (Stages not defined)	GFR, proteinuria, renal function, anthropometry/nutritional status, compliance with diet, hyperglycemia, insulin requirements	Patients with insulin dependent diabetes. Interventions of nutritional support with amino acid or keto-acid supplements with or without restriction of protein intake	Protein restricted diets at least <0.8–1 g/kg/day is only recommended in Type 1 DM, showing reduction in hyperglycemia and decreased insulin requirements. Anthropometric parameters were preserved. LPD (using specific enteral supplements) should be used by patients in the early stages of diabetic nephropathy to slow progression of renal failure. No specific protein intake levels are prescribed.	I
**Weight loss (patients with and without diabetes)**						
Navaneethan [25]	13 studies (2 RCT and 11 observational)	*n* = 520 (174 non-surgical interventions, 346 surgical interventions) Stages 1–4	Renal function (GFR or creatinine clearance, proteinuria). Anthropometry (BMI *). Biochemistry (HbA1C *, serum lipids). Other (Blood Pressure)	Obese patients (BMI ≥ 30). RCTs or observational studies of surgical or non-surgical weight loss interventions among patients with either existing CKD or obesity-related glomerular hyperfiltration. Follow up of ≥4 weeks.	Non-surgical weight loss did not elicit change in GFR or creatinine clearance, but was associated with a reduction in proteinuria, BMI, Systolic BP * and Total cholesterol. Surgical weight loss was associated with normalisation of GFR in glomerular hyperfiltration, significant reduction in BMI, proteinuria and systolic BP. Only modest evidence to support the role of intentional weight loss in slowing CKD progression in mild-moderate CKD	I
Enteral support						
Stratton [26]	18 studies (5 RCT and 13 non-RCT)	Maintenance HD *, Stage 5	Clinical: QoL*, Complications, mortality Biochemical: albumin and electrolyte levels Nutritional: dietary intake, anthropometry	Multi-nutrient oral supplements and enteral tube feeding which included nutrition support (NS) with routine care; disease specific formula with standard formulae; enteral feeding with parenteral feeding.	Enteral feeding *vs.* routine care increased energy and protein intake and increased serum albumin concentration by0.23 g/dL (2.3 g/L: 95% CI * 0.037 to 0.418 g/dL. There was insufficient data to examine the effect of this on clinical outcomes. Additional research required, especially comparing disease specific formulae with standard formulae.	I

* LPD, Low protein diet; * CRF, Chronic Renal Failure; * GFR, Glomerular Filtration Rate; * RCT, Randomised Controlled Trial; * VLPD, Very low protein diet; * QOL, Quality of Life; * DM, Diabetes Mellitus; * CKD Chronic Kidney Disease; * UAER, Urinary Albumin Excretion Rate; * BMI, Body Mass Index; * BP, Blood Pressure; * HbA1C, Glycosylated Haemoglobin; * HD, Haemodialysis; * CI Confidence Interval.

**Table 2:** Experimental and observational studies addressing various aspects of the nutrition prescription in CKD.

Author	Study Design and Length	Sample Characteristics	Intervention	Outcomes	Results/Conclusions	Level of Evidence [20]
**Protein—experimental studies**						
Feiten [31]	RCT * (4 months)	*n = 24* Pre-dialysis (Stage 4 and 5) >18 years	Intervention: VLPD * (0.3 g vegetal protein/kg IBW */day) + KA * Control: LPD (0.6 g protein/kg IBW/day)	Nutrient intake & compliance (3 day food diary, normalised protein appearance (nNPA *)). Anthropometry (BMI *, %TSF *, % MAMC *, LBMI *). Serum and urinary urea, serum creatinine, ionised calcium, bicarbonate, albumin, iPTH, eGFR *)	Nutritional status was maintained but compliance was poor in both groups. Protein intake was underestimated by approximately 28% in both groups when food records and nNPA were compared. Actual protein intake of intervention group decreased significantly from 0.9 ± 0.24 g/kg/day to 0.66 ± 0.11 g/kg/day at 4 months (*p* < 0.05) while energy remained stable (22.9 kcal/kg/day in VLPD * and 24 kcal/kg/day in LPD. Serum urea nitrogen from 61.4 to 43.6 mg/dL, *p* < 0.001. Dietary PO4 * decreased, with improvements in Ca * and PTH * metabolism.	II
Cianciaruso [32]	Follow up data from a RCT (48 months)	*n* = 423 Pre-dialysis (stage 4 and 5)	Intervention: LPD * (0.55 g/kg/dat) Control: MPD * (0.8 g/kg/day)	Protein energy malnutrition; Progression to dialysis; Mortality; Composite end point (death or dialysis)	Protein intakes were 0.73 ± 0.04 g/kg/day for LPD and 0.9 ± 0.06 g/kg/day for MPD. Unadjusted Cox survival analyses were 1.01 (95% CI * 0.57–1.79) 0.90 (95% CI 0.62–1.48) and 0.98 (95% CI 0.68–1.43) respectively for death, progression to dialysis or composite end point with no differences in outcome of either intervention.	II
Brunori [28]	RCT (1 year)	*n* = 112 (Stage 5 GFR 5–7 mL/min)	Intervention: LPD (0.3 g/kg/day, 35 kcal/day + ketoacids, vitamins, minerals. Control: Dialysis	Mortality, hospitalization, metabolic markers	Median follow-up was 26.5 months (IQR *, 40). Patients in diet group spent median 10.7 months on VLPD (IQR *, 11). 31 deaths (55%) in the dialysis group; 28 deaths (50%) in the diet group. One-year observed survival rates at intention to treat 83.7% (95% CI, 74.5 to 94.0) dialysis group *versus* 87.3% (95% CI, 78.9 to 96.5) diet group; difference in survival −3.6% (95% CI, −17 to −10; *p* = 0.002). The hazard ratio for hospitalization was 1.50 for the dialysis group (95% CI, 1.11 to 2.01; *p* < 0.01).	II
**Protein—observational studies**						
Vendrely [29]	Comparative study with con-current controls, 12 months	*n* = 30 Dialysis (Stage 5, HD *)	Intervention group: VLPD (0.3 g/kg/day supplemented with essential amino acids, Calcium, Iron and vitamins) prior to initiation of HD. Control: Less restrictive diet (~0.9 g/kg/day) prior to initiation of HD.	Nutrient intake (3 day food record every 3 months). Anthropometry (BMI, body composition by DEXA). Serum albumin and pre-albumin.	Protein intake increased to >1.2 g/kg/day, BMI increased by 0.97 ± 1.31 kg/m^2^, *p* < 0.001, due to increased in fat mass 2.36 ± 2.94 kg/m^2^, *p* < 0.001 in both groups 3 months after commencement of HD. No differences were observed between groups for LBM, BMI, serum albumin or pre-albumin.	III-2
Kanazawa [33]	Comparative study with concurrent controls (not randomised)	*n* = 65 Pre-dialysis (Stages 3–5)	Case group: Non-compliant on LPD (0.69 g/kg/day) > 3 months. Control group: Compliant on LPD (0.69 g/kg/day) > 3 months	Biochemistry (GFR, serum creatinine, BUN *, reciprocal of serum creatinine). Dietary compliance (3 day food records, PCR *). Health related QOL *	Change in mean GFR rate was lower in compliant group (−0.063 ± 1.306 compared to −0.742 ± 1.18, *p* < 0.05. No difference between groups for health-related QOL.	III-2
Shinaberger [30]	Retrospective cohort study. 2 years	*n* = 53,933 Dialysis (Stage 5, Maintenance HD)	Historical review of maintenance HD patients’ protein intake (measured by nPNA and categorised into 10 increments) & mortality	Protein intake ( measured by nPNA) MICS * (malnutrition-inflammation complex syndrome) All-cause mortality Cardiovascular mortality	Hazard ratios were not significantly increased with nPNA between 1–1.4 g/kg/day but increased to 1.34 (95% CI 1.23–1.46, *p* < 0.0001), when levels were <0.6 or ≥1.4 g/kg/day. Protein intakes of <0.8 or >1.4 g/kg/day associated with greater mortality, even when adjusted for MICS and case mix. Increasing protein intake of patients in the 0.8–1.2 g/kg/day protein range within the first 6 months, tended to reduce mortality risk, whilst a decreased protein intake in the first 6 months, increased the risk.	III-3
Chauveau [27]	Prospective cohort study no concurrent controls, 5 years	*n* = 203 Predialysis (Stage 4–5)	VLPD (0.3 g protein/kg/day, 35 kcal/day, 5–7 mg phosphate + ketoacids) for >3 months	Mortality; Progression to dialysis or transplant	Mean duration of diet period 33.1 months (4–230). Overall survival rate 79% and 63% at 5 and 10 year, respectively. 102 patients continued with chronic dialysis during the entire follow-up, and 101 patients were grafted at least once. No correlation between death and duration of diet.	III-3
**Vitamin D-experimental studies**						
Fishbane [34]	RCT (double blind, 6 months	*n* = 61 Pre-dialysis (Stages 1–4).	Intervention: oral paricalcitol, 1 μg/day Control: placebo	Biochemistry (mean spot urinary protein-creatinine ratio, serum intact PTH, serum calcium, serum phosphorus, urine creatinine)	Significant decrease in proteinuria in paricalcitol group. Mean spot urinary protein-creatinine ratios were +2.9% in controls and −17.6% in the intervention group (*p* = 0.04). Serum iPTH ↓significantly in intervention group (*p* = 0.01). 57.6% of paricalcitol group had a more than 10% decline in proteinuria. Modest effect size noted as is small study size.	II
Agarwal [35]	RCT (double blind, 24 weeks	*n* = 220 Pre-dialysis (Stage 3–4) Secondary hyperparathyroidism	Intervention: oral paricalcitol 9.5 μg/week Control: placebo	Proteinuria	51% intervention group compared to 25% control reduced proteinuria (OR 3.2, 95% CI 1.5–6.9, p = 0.004). For those with proteinuria and PTH suppression (2 consecutive ≥30% decrease in iPTH from baseline) proteinuria decreased 53% intervention vs. 0% in control.	II
**Vitamin D—observational studies**						
Wang [36]	Cohort study (prospective), 3 years	*N* = 230 Dialysis (Stage 5, PD *)	Serum Vitamin D (25(OH)D) and clinical outcomes (death, fatal cardiovascular event, non-fatal cardiovascular event)	Anthropometry (BMI) Serum 25(OH)D, eGFR echocardiography Nutritional status (SGA *) Dialysis adequacy All cause mortality Cardiovascular events (fatal or non-fatal)	87% of cohort were deficient or insufficient in 25(OH)D (<75 nmol/L). Kaplan Meier estimates show a significantly greater fatal or non-fatal CV * event-free survival probability in patients whose 25(OH)D >median 45.7 nmol/L than those with median ≤45 nmol/L (*p* = 0.004).	III-2
**Fats–experimental studies**						
Beavers [37]	RCT (double blind, permuted-randomised), 6 months	*n* = 69 Dialysis (Stage 5, HD)	Intervention: daily supplement of 6 g *n*-3 fatty acids in the form of fish oil (160 mg EPA *, 100 mg DHA *) Control:6 g daily supplement corn oil (*n*-6)	Biochemistry (total homocysteine) Compliance: Pill counting (NB did not use *in vivo* testing)	Over the counter omega-3 fatty acids at 6 g per day have no effect on total homocysteine compared to a placebo.	II
**Fats—observational studies**						
Saltissi [38]	Case series, 14 weeks	*n* = 75 Dialysis (Stage 5, HD and PD) with dyslipidaemia	Dietary prescription: Adjustment of “dialysis diet” to bring in line with Australian NHF * guidelines to reduce lipid levels for chronic PD and HD patients	Anthropometry (BMI). Nutrient intake: Dietary assessment and computer analysis, Biochemistry (total, HDL * , LDL *, VLDL * cholesterol, TG *)	In HD patients, decreased saturated fat and cholesterol intake was associated with a decrease in total cholesterol (*p* = 0.007) and LDL cholesterol (*p* < 0.01) but not in PD. Most dialysis patients will require pharmacologic lipid lowering treatment for adequate control.	IV
**Sodium and fluid—experimental studies**						
Vogt [39]	RCT (double blind, placebo controlled crossover), 36 weeks	Patients with proteinuria (various diagnoses)	Intervention: Treatment with placebo, Losartan, Losartan + HCT * whilst randomised to either high sodium (200 mmol/day) *vs.* low sodium (50 mmol/day) diet.	Anthropometry (BMI). Biochemistry (proteinuria, serum creatinine, urea, cholesterol, triglycerides, total protein and albumin). Other (urinary sodium excretion, mean arterial pressure, systolic and diastolic blood pressures)	Baseline proteinuria was decreased by 22% by LSD * alone, Losartan decreased proteinuria by 30%, Losartan + LSD decreased proteinuria by 55%. The combined addition of HCT and low-sodium diet decreased proteinuria by 70% from baseline (all *p* < 0.05). Reductions in mean arterial pressure showed a similar pattern (all *p*< 0.05). A low sodium diet and HCT are equally efficacious in reducing proteinuria and BP when added to a regimen containing Losartan and especially seem to benefit individuals in whom proteinuria is resistant to Renin-Angiotensin-Aldosterone system blockade. Sodium restriction exerted a modest but significant antiproteinuric effect.	II
**Sodium and fluid—observational studies**						
Kayikcioglu [40]	Retrospective cross sectional study comparing 2 centres, I year	*n* = 394 Stage 5, HD. Centre A (*n* = 190)—salt restriction. Centre B (*n* = 204)—hypertensive drugs	Intervention: salt restricted diet (5 g/day) and intensive ultrafiltration to maintain pre-dialysis B, *P* < 140/90 mmHg without antihypertensive medication. Control: Hypertensive drugs	Hypertensive drug use. Weight and BP. Systolic and diastolic function. Intradialytic hypotension	Antihypertensive drugs used in 7% Centre A and 42% in Centre B (*p* < 0.01); Interdialytic weight gain was significantly lower in Centre A (2.29 ± 0.83 kg *vs.* 3.31 ± 1.12 kg, *p* < 0.001). Mean systolic and diastolic BP similar. Frequency of LV hypertrophy was lower in Centre A (74% *versus* 88%, *p* < 0.001). Intradialytic hypotension (hypotensive episodes/100 patient sessions) was more frequent in Centre B (11 *versus* 27, *p* < 0.01).	III-2
Boudville [41]	Retrospective cohort, 5 years	*n* = 141. (Stages 4–5, including dialysis + 24 h urine collection for sodium)	24 h sodium excretion divided into tertiles. Percentiles 33.3 and 66.6 being 114.0 mmol/day Na. (2.7 g/day) and 166.7 mmol/day Na (4.0 g/day), respectively	Hypertensive drug use. BP control	Mean (±SE) sodium excretion 145.7 ± 4.7 mmol/day (3.5 g Na/day). Control of BP equivalent in all groups. Greater no. antihypertensive agents with increased sodium excretion (2.00 ± 0.16, 2.61 ± 0.20, and 2.77 ± 0.19 medications, respectively for each tertile; *p* = 0.01). For those with GFR ≤ 15 mL/min (*n* = 77) medications used with increased sodium excretion 1.69 ± 0.19, 2.52 ± 0.27, and 3.08 ± 0.26, respectively; *p* = 0.001. Multivariable analysis sodium excretion (*p* = 0.00005) and age (*p* = 0.007) significantly associated with use of antihypertensive medication.	III-3
**Fibre—experimental studies**						
Sutton [42]	Interrupted time series without parallel control group	Stage 1: *n* = 126 Stage 2: 4 weeks: *n* = 23 Stage 3: 3 weeks: *n* = 17 Dialysis (Stage 5, PD) regularly using laxatives	Stage 1: Survey Stage 2: laxative users replaced laxatives with 6–12 g/day partially hydrolysed guar gum supplement Stage 3: dietary counselling to support increased dietary fibre intake of 6–12 g/day from foods	Patient reported preference for efficacy, ease of administration, acceptability of taste and texture for laxative, supplement or increased dietary fibre.Self reported bowel habits (Bristol stool chart) Laxative use	Of 23 patients involved in intervention, 15 thought the fibre supplement provided best stool result and reduced side effects and 14 preferred the supplement over laxative. No objective data reported. Poor quality study, as reported outcomes were not matched objectively against fibre intake.	IV
**Oral nutrition supplements—experimental studies**						
Teixido-Planas [43]	Open RCT (multicentre), 12 months	*n* = 65. Dialysis (Stage 5, PD)	Intervention: 200 mL (1.0 kcal/mL) liquid protein supplement daily in addition to normal dietary intake. Control: no protein supplement, usual dietary intake	Nutrient intake (3 day food record). Anthropometry (BMI, skinfolds, BSA *). Nutritional status (SGA). Biochemistry (full blood count, serum albumin, lymphocyte count, lipids, urea, creatinine). Clinical (dialysis adequacy, urinary and peritoneal losses). Patient compliance (patient report, family report, inventory check).	Intention to treat analysis revealed a significant improvement in the intervention group in lymphocyte count (*p* *<* 0.001), weight (*p* < 0.03), TSF (*p* < 0.001), MAMC * (*p* *<* 0.005). The supplement used was not suitable for long term use due to a high rate of non-compliance and high dropout in the intervention. Malnutrition assessed by SGA decreased from 29% in intervention to 0% and from 33% in controls to 20%.	II
Caglar [44]	Pilot prospective cohort study, 9 months, with 3 months baseline	*n* = 85. Dialysis (Stage 5, HD + diagnosed malnutrition)	Intervention: 200 mL (2.0 kcal/mL) liquid protein supplement during dialysis treatment, 3 to 9 months. Control: standard nutritional counselling, baseline to 3 months	Nutrient intake (48 h dietary recall). Anthropometry (BMI). Biochemistry (albumin, pre-albumin, transferrin). Nutritional status (SGA)	ONS * improved nutritional parameters (significant increase in serum albumin (3.33 ± 0.32 g/dL baseline to 3.65 ± 0.26 g/dL end 6 month intervention, *p* < 0.0001), serum pre-albumin (26.1 ± 8.57 g/dL baseline to 30.7 ± 7.36 g/dL end 6 month intervention, *p* < 0.0001) and SGA score (4.94 ± 1.23 g/dL baseline to 5.64 ± 0.90 g/dL end 6 month intervention, *p* < 0.05)). BMI and body weight increased non-significantly from baseline to end of intervention. Note: High non-compliance rate (32%). Less than half of participants completed the study (46%).	III-2
**Oral nutrition supplements—experimental studies**						
Gonzales-Espinoza [45]	Open RCT, 6 months	*n* = 28. Dialysis (Stage 5, PD)	Intervention: nutritional counselling + 30 g oral egg-albumin protein supplement of 22 g protein/day. Control: nutritional counselling.	Nutrient intake (24 h recall). Anthropometry (BMI, skin folds).Biochemistry (serum albumin, creatinine, lipids, nPNA, glucose, BUN *). Other (dialysis adequacy).Patient compliance (weighed inventory of supplement).	Frequency of moderate-severe malnutrition decreased 28% in intervention group (*vs*. 6% in control group). Comparing baseline to 6 months, ONS significantly improved serum albumin (2.64 ± 0.35 *vs.* 3.05 ± 0.72 g/dL) and energy intake (1331 ± 342 *vs.* 1872 ± 698 kcal/day) in the same group, *p* < 0.05 and protein intake (1.0 ± 0.3 *vs.* 1.7 ± 0.7 g/kg) and nPNA (1.00 ± 0.23 *vs.* 1.18 ± 0.35 g/kg/day) within and between groups (*p* < 0.05) with a trend to increased anthropometric parameters and nutritional status in the intervention group. Multivariate analysis showed only serum albumin significantly predicted by ONS (β 0.72, 95% CI 0.14–1.3, *p* = 0.02) and % protein intake (β −0.01, 95% CI (0–0.02, *p* = 0.05) and SGA significantly predicted by TSF (RR 0.79, 95%CI 0.63−0.98, *p* = 0.03. Compliance was high at 90%.	II
**Nutritional Counselling—intervention studies**						
Campbell (2008) [46]	RCT (12 weeks)	*n* = 56. Pre-dialysis (Stage 4 and 5)	Intervention: Regular and individualised dietary counselling. Control: written nutrition education material	Nutrient intake (3 day food record). Anthropometry (body composition). Nutritional status (SGA)	Intervention group had a 3.5% (95% CI −2.1 to 9.1), less decrease in body cell mass, 17.7 kJ/kg/day (95% CI 8.2 to 27.2) increase in energy intake, greater improvement in SGA, all *p* < 0.01 and no significant increase in protein intake. Structured nutrition intervention had a greater effect on energy and protein intake in women than men (interaction *p* < 0.001 for both).	II
Campbell (2008) [47]	RCT (12 weeks)	*n* = 53. Pre-dialysis (Stage 4 and 5)	Intervention: Regular and individualised dietary counselling. Control: written nutrition education material	Nutritional status (PG-SGA *). KDQoL *	Intervention showed significant improvement in subscales of KDQoL compared to nutritional status: symptoms 7.1 (0.1–14.1), *p* = 0.047; cognitive functioning 14.6 (5.4–23.7), *p* = 0.03; vitality 12.0 (4.6–19.5) *p* *=* 0.002.	II
**Nutritional Counselling—intervention studies**						
Sullivan, Sayre *et al.* 2009 [48]	Cluster RCT, 14 facilities, 2 shifts at 12 large centres and 1 shift at 2 small centres, 3 months	*n* = 279. HD (Stage 5). Intervention *n* = 145: Control *n* = 134	Intervention: education on avoiding food with PO_4_ * additives. Control: Usual care. 3 month duration	Change in serum PO_4_	Intervention gp showed decrease in serum PO_4_ of −0.6 mg/dL (95% CI −1.0 to −0.1 mg/dL, *p* = 0.03). This change was not explained by change in food knowledge score but intervention group showed significant improvements in reading nutrition facts label score 9 (95% CI 1 to 17, *p* = 0.04) and food ingredients list score 22 (95% CI 15–30 *p* *<* 0.001).	II
Morey, Walker *et al*. [49]	RCT, 6 months	*n* = 67 stable HD (Stage 5)	Intervention: Monthly dietetic counselling to improve PO_4_ intake and binder adherence. Control: 6 month counselling	Change in serum PO_4,_ controlling for serum PO_4_, binder use and alphacalcidrol at baseline	Intervention group showed decrease in serum PO_4_ at 3 months approaching significance when controlled for confounders—0.253 mg/dL (95% CI −0.513 to 0.007 mg/dL, *p* = 0.056) compared to control however this difference disappeared at 6 months.	II
**Nutritional Counselling—observational studies**						
Campbell (2009) [50]	Retrospective observational study, 2 years with 3 time points	*n* = 65. Dialysis (Stage 5, maintenance HD)	Dietary interview (at least every 6 months with intensive follow up where required).	Nutrient intake (dietary interview). Anthropometry (serum albumin and potassium). Biochemistry. Nutritional status (SGA)	Proportion of patients with malnutrition (as per SGA) decreased from 14% to 3% after 2 years. Serum albumin, potassium and dry weight remained stable. Significant decrease in serum phosphate (mean ± SD, 1.8 ± 0.5 to 1.5 ± 0.5 mmol/L, *p* = 0.004). Energy intake increased to 105 kJ/kg from 102 kJ/kg at baseline (*p* = 0.001) and protein intake increased from 1.14 g/kg/day to 1.18 g/kg/day (*p* = 0.022). Under-reporting occurred in 30%–60% patients.	III-3
**Nutrients in peritoneal dialysis solution—experimental studies**						
Tjong (2005) [51]	Randomised cross-over study, 14 days	*n* = 8. Dialysis (Stage 5, PD)	Intervention: AAPD * (plus glucose). Control: Standard PD solution	Biochemistry (WBPT *, 24 h nitrogen balance)	Net protein balance (protein synthesis minus protein breakdown) increased on AA PD in all patients (mean 0.21 ± 0.12 μmol leucine/kg per min; *p* < 0.001). The 24-h nitrogen balance changed by 0.96 ± 1.21g/day, from −0.60 ± 2.38 to 0.35 ± 3.25 g/day (*p* * =* 0.061, NS), improving in six patients.	II
**Nutrients in peritoneal dialysis solution—observational studies**						
Sezer (2006) [52]	Prospective, open labelled uncontrolled study, 3 months	*n* = 16. Dialysis (Stage 5, PD) with hypoalbuminaemia	Amino acid peritoneal dialysis (AAPD). 1 Dextrose peritoneal dialysate exchange/day replaced by a 2 L AAPD bag.	Anthropometry (LBM *). Biochemistry (albumin, lipids). Nutritional status (SGA)	Albumin improved 3.5 ± 0.5 g/dL to 4.1 ± 0.4 g/dL (*p* = 0.003); HDL cholesterol level decreased from 43.1 ± 7.3 mg/dL to 37.8 ± 6.0 mg/dL (*p* = 0.02), even though other lipid parameters (total cholesterol, triglyceride and LDL cholesterol) did not change.	IV
**Intradialytic Parenteral Nutrition—experimental studies**						
Pupim (2004) [53]	Randomised prospective cross over study	*n* = 7. Dialysis (Stage 5, HD)	IDPN *	Biochemistry (albumin fractional synthetic rate, WBPT *)	Nutritional supplementation in the form of IDPN improves the hepatic synthesis of albumin (16.2 ± 1.5%/day *vs.* 12.8 ± 1.7%/day, *p <* 0.05) as a part of improvements in the whole body protein synthesis (5.05 ± 0.3 mg/kg fat-free mass/min *vs*. 3.22 ± 0.3 mg/kg fat-free mass/min (*p* < 0.05).	II
Pupim (2006) [54]	Randomised prospective cross over study	*n* = 8. Dialysis (Stage 5, HD)	Intervention: IDPN or oral nutritional supplement during HD treatment. Control: normal HD treatment	Biochemistry (albumin, prealbumin, transferrin, metabolic hormones, serum protein, *etc.*)	Positive whole-body net balance during HD with both IDPN and ONS, 4.43 ± 0.7 and 5.71 ± 1.2 mg/kg fat-free mass per min, respectively, compared with control (0.25 ± 0.5 mg/kg fat-free mass per min; *p* =0.002 and <0.001) for IDPN *versus* control and ONS *versus* control, respectively. ONS resulted in persistent anabolic benefits in the post-HD phase for muscle protein metabolism, when anabolic benefits of IDPN dissipated.	II
**Intradialytic Parenteral Nutrition—observational studies**						
Cherry (2002) [55]	Case series, 12 months	*n* = 24. Dialysis (Stage 5, PD). Malnourished, using criteria	Intervention: 2 formulations 750 mL and 1000 mL IDPN, both 925 non protein calories, 1000-mL formulation provided an extra 25 g of protein.	Anthropometry (dry body weight). Biochemistry (serum albumin)	Body weight increased from median 46.8 kg at baseline to 47.5 at 6 months and 53.8 at 12 months (*p* *<* 0.05, *p* < 0.05, *p* < 0.003 respectively). Serum albumin levels increased from median of 27.5 at baseline to 31.0 at 3 months (*p* *<* 0.05) and 30.5 at 12 months in malnourished HD patients. Significant attrition at 9 and 12 months (*n* = 16)	IV
**Intradialytic Parenteral Nutrition—observational studies**						
Joannidis (2008) [56]	Prospective cohort study with matched controls, 6 months	*n* = 12. Dialysis (Stage 5, PD) with MICS. Controls had no malnutrition	Intervention: IDPN 100 mL glucose 60%, 100 mL Elolipid 20% (soya bean oil 100 g/1000 mL, glycerol 25 g/1000 mL, egglecithin 12 g/1000 mL Control: usual dialysis	Anthropometry (weight, BMI)Biochemistry (lipids, inflammatory markers	Mean body weight increased from 61.7 ± 7.7 to 63.9 ± 8.9 kg (*p* = 0.03) and BMI increasred from 21.9 ± 3.4 to 22.8 ± 3.9 kg/m^2^, *p* = 0.03, compared to no change in control group. nPCR values differed significantly between patients at baseline but no significant difference was observed at the completion of the study for any other biochemical or nutritional markers.	III-2
Korzets (2008) [57]	Prospective observational case series, 1.5 to 17 months	*n* = 22. Dialysis (Stage 5, HD)	IDPN: Total E 1174–1677 kcal; Amino acids 10% 50–85 g; dextrose 50% 125–185 g; Clinoleic 20% 50–70 g, following major surgical or medical illnesses	Anthropometry. Biochemistry (protein catabolic rate, albumin, pre-albumin, creatinine). Dialysis adequacy	nPCR increased from 0.7 ± 0.2 to 1.2 ± 0.2 g protein/kg/ day (*p* *<* 0.0001); serum albumin increased from 28 ± 5 g/L to 38 ± 2 g/L (*p* < 0.0001); serum pre-albumin levels increased from 210 ± 82 to 300 ± 52 mg/L (*p* < 0.01 and serum creatinine increased from 504 ± 195 to 672 ± 186 μmol/L (*p =* 0.016). Serum cholesterol increased from 3.5 ± 1.4 to 4.4 ± 1.4 mmol/L (*p* < 0.0001). Kt/V levels and weight did not change significantly during IDPN (1.43 ± 0.22 to 1.46 ± 0.26).	IV
**Percutaneous Endoscopic Gastrostomy (PEG) feeding**						
(Sayce 2000) [58]	Case series. Pre and post intervention over 3 months	*n* = 8. Dialysis (Stage 5, HD). +malnutrition	Various PEG feeding regimens; E 1983–7205 kcal/day; Pro 17–61 g/day	Anthropometry (weight, skin folds). Biochemistry (albumin). Cost (hospitalisations and complications)	Median dry weight increased from 43 to 48.3 kg (*p* = 0.012); BMI increased from 16.4 to 18.8 kg/m^2^ (*p* = 0.012); MUAC increase from 20.2 to 24.8 cm (*p* = 0.018); TSF increased from 7.3 to 11.3 mm (*p* = 0.046); MUAMC increased from 17.7 to 19.8 cm (*p* *=* 0.027); Serum albumin increased from 29.5 to 36.5 g/L (*p* = 0.011)	IV

* CKD Chronic Kidney Disease; * HD Haemodialysis; * PD Peritoneal Dialysis; * GFR Glomerular Filtration Rate; * RCT Randomised Controlled Trial; * MPD Moderate protein diet; * LPD Low protein diet; * VLPD Very low protein diet; * KA Keto-acids; * NHF National Heart Foundation; * LSD Low sodium diet; * HSD High sodium diet, * BUN Blood urea nitrogen; * QOL quality of life; * POM profile of mood states; * BSA body surface area; * iPTH intact parathyroid hormone; * AAPD Amino acid peritoneal dialysate; * LBM Lean body mass; * WBPT Whole body protein turnover; * IDPN Intra-dialytic parenteral nutrition; * MICS malnutrition-inflammation complex syndrome; * WBPT Whole body protein synthesis; * UAER Urinary Albumin Excretion Rate; * PCR Protein Catabolic Rate; * nNPA Normalised Protein Appearance; * CRP c-reactive protein; * SGA Subjective Global Assessment; * PG-SGA Patient Generated Subjective Global Assessment; * Hb Haemoglobin; * HDL High density lipoprotein; * LDL low density lipoprotein; * VLDL very low density lipoprotein, TG Triglyceride; * PO_4_ phosphate; * HCT hydrochlorothiazide; * MUAC Mid Upper Arm Circumference; * TSF Triceps Skinfold Thickness; * MUAMC Mid Upper Arm Muscle Circumference; * MAMC Mid Arm Muscle Circumference; * BMI Body mass index; * EPA Eicosopentanoic Acid; * DHA Decosahexanoic Acid; * Ca Calcium; * ONS Oral Nutrition Support; * CV Cadiovascular; * KDQoL Kidney Disease Quality of Life; * CI Confidence Interval; * Na sodium; * LV left ventricular; * ESRD End Stage Renal Disease; * IQR interquartile range.

**Table 3:** Nutritional Parameter in International Guidelines with evidence.

Nutrient or Requirement	Most Current Equivalent Guideline Statement	Grade of Evidence Equivalent to GRADE [59]
Energy-dialysis	**KDOQI (2000) [60], BDA (2013) [19]**	
	The recommended daily energy intake for maintenance haemodialysis or chronic peritoneal dialysis patients is 35 kcal/kg ideal body weight/day (146 kJ/kg IBW/day) for those who are less than 60 years of age and 30 to 35 kcal/kg body weight/day (126–146 kJ/kg IBW/day) for individuals 60 years or older.	C
Protein–pre-dialysis	**CARI (2013) [15]**	
	We recommend for patients with early CKD consume a normal protein diet of 0.75–1.0 g/kg IBW/day with adequate energy. This is the Recommended Dietary Intake for the general population.	1C
	A low protein diet (≤0.6 g/kg IBW/day) to slow down CKD progression is not recommended because of the risk of malnutrition.	1C
	We suggest that patients with excess protein intakes reduce their intakes to the RDI levels as a high protein diet may accelerate renal function decline in mild renal insufficiency	2C
Protein–pre-dialysis with keto acids	**ADA (2010) [18]**	
	For adults with CKD without diabetes, not on dialysis, with an eGFR < 20 mL/min, a very low protein controlled diet providing 0.3 g–0.5 g dietary protein per kg of body weight per day with addition of keto acid analogs to meet protein requirements may be recommended. International studies report that additional keto acid analogs and vitamin or mineral supplementation are needed to maintain adequate nutrition status for patients with CKD who consume a very low protein controlled diet (0.3–0.5 g/kg/day)	Strong, conditional evidence
Protein-dialysis	**KDOQI (2000) [59] BDA (2013) [19]**	
	The recommended dietary protein intake for clinically and weight stable maintenance HD patients is 1.1 g/kg ideal body weight/day. At least 50% of the dietary protein should be of high biological value. For clinically and weight stable PD patients, the recommended protein intake is 1.0–1.2 g/kg ideal body weight/day. Those who are not stable may need higher levels of protein.	C
Sodium-pre-dialysis	**CARI (2013) [15]**	
	We recommend that early CKD patients restrict their dietary sodium intake to below 100 mmoL per day or less, as it reduces blood pressure and albuminuria in patients with CKD.	1C
Sodium-dialysis	**KDOQI (2000)[59]**	
	Dietary sodium intake of less than 2.4 g/day (less than 100 mmol/day) should be recommended in most adults with CKD and hypertension.	A
Fluid-pre-dialysis	**CARI (2013) [15]**	
	We suggest that patients drink fluids in moderation. For most patients with early CKD, a daily fluid intake of 2–2.5 L (including fluid content of foods) is sufficient, although this may need to be varied for individual circumstances.	2C
Phosphate-pre-dialysis	**CARI (2013) [15]**	
	We suggest that early CKD patients (stages 1–3) should not restrict dietary phosphate intake as restrictions of dietary phosphate does not influence renal or cardiovascular outcomes in these patients.	2C
	**KDIGO (2009) [17]**	
	In patients with CKD stages 3–5, we suggest maintaining serum phosphorus in the normal range.	2C
	In patients with CKD stages 3–5 we suggest using phosphate-binding agents in the treatment of hyperphosphatemia.	2D
	It is reasonable that the choice of phosphate binder takes into account CKD stage, presence of other components of CKD–MBD, concomitant therapies, and side-effect profile.	Not graded
Phosphate-dialysis	**KDIGO (2009) [17]**	
	In patients with CKD stage 5D, we suggest lowering elevated phosphorus levels toward the normal range.	2C
	In patients with CKD stages 5D we suggest using phosphate-binding agents in the treatment of hyperphosphatemia.	2B
	It is reasonable that the choice of phosphate binder takes into account CKD stage, presence of other components of CKD–MBD, concomitant therapies, and side-effect profile.	Not graded
	In patients with CKD stages 3–5D and hyperphosphatemia, we recommend restricting the dose of calcium-based phosphate binders and/or the dose of calcitriol or vitamin D analog in the presence of persistent or recurrent hypercalcemia.	1B
	In patients with CKD stages 3–5D and hyperphosphatemia, we suggest restricting the dose of calcium based phosphate binders in the presence of arterial calcification and/or adynamic bone disease and/or if serum PTH levels are persistently low.	2C
	In patients with CKD stages 3–5D, we recommend avoiding the long-term use of aluminum-containing phosphate binders and, in patients with CKD stage 5D, avoiding dialysate aluminum contamination to prevent aluminum intoxication.	1C
	In patients with CKD stages 3–5D, we suggest limiting dietary phosphate intake in the treatment of hyperphosphatemia alone or in combination with other treatments.	2D
Fibre	**CARI (2103) [15]**	
	We suggest patients with early CKD consume a diet rich in dietary fibre that is associated with reduced inflammation and mortality in CKD patients.	2D
Potassium-pre-dialysis	**CARI (2013) [15]**	
	We suggest that early CKD patients with persistent hyperkalaemia restrict their dietary potassium intake with the assistance of a qualified dietitian.	2D
Vitamin D-pre-dialysis	**CARI (2013) [15]**	
	We suggest Vitamin D deficiency (25 hydroxy vitamin D < 37.5nmol/L) and insufficiency (25 hydroxy vitamin D 35.5–75 nmol/L) if present be corrected using treatment strategies for the general population:	2C
	Daily oral intake 19–50 year: 5 μg; 51–70 year: 10 μg; >70 year: 15 μg (1 μg = 40 IU). It is very difficult to meet RDI with food intake alone.	2D
	A few minutes in Australian summer for fair skinned people and 2–3 h of sunlight/week in winter in southern regions.	2D
	We recommend a prescription of vitamin D therapy for early CKD patients with secondary hyperparathyroidism, as it has been shown to be effective in suppressing elevated levels of parathryroid (PTH) hormone. There is insufficient evidence to determine whether this improves patient-level outcomes and the potential benefits of vitamin D therapy must be weighed against its potential deleterious effects, including hypercalcaemia, hyperphosphataemia, vascular calcification, adynamic bone disease and accelerated progression of CKD.	1A
	We recommend that early CKD patients on vitamin D therapy have their calcium, phosphate, PTH, alkaline phosphate and 25(OH) vitamin D level monitored regularly.	1C
Vitamin D-dialysis	**KDIGO (2009) [17]**	
	In patients with CKD stage 5D and elevated or rising PTH, we suggest calcitriol, or vitamin D analogs, or calcimimetics, or a combination of calcimimetics and calcitriol or vitamin D analogs be used to lower PTH.	2B
Calorie restriction/weight loss	**CARI (2013) [15]**	
	We recommend that overweight/obese patients with CKD should be prescribed caloric restriction under the management of an appropriately qualified dietitian. A reduction in weight can mean an improvement of CKD.	1C
	We suggest, in the absence of specific recommendations for CKD, overweight and obese patients are encouraged to aim for a body mass index (BMI) of between 18.5 and 24.9 kg/m^2^ and waist circumference of ≤102 cm for men and ≤88 cm for women.	2C
	**CMA (2008) [11]**	
	Obese (BMI > 30.0 kg/m^2^) and overweight (BMI 25.0–29.9 kg/m^2^) people should be encouraged to reduce their BMI to lower their risk of chronic kidney diseaseand end-stage renal disease.	D
	Maintenance of a health body weight (BMI 18.5–24.9 kg/m^2^; waistcircumference < 102 cm for men, <88 cm for women) is recommended to prevent hypertension.	C
	Or to reduce blood pressure in those with hypertension.	B
	All overweight people with hypertension should be advised to lose weight.	B
Other dietary components	**CARI (2013) [15]**	
	Fruit and vegetables—we suggest adults with early CKD consume a balanced diet rich in fruit and vegetables, as these appear to reduce blood pressure and have renoprotective effects comparable to sodium bicarbonate.	2C
	Mediterranean diet—we suggest adults with CKD consume a Mediterranean style diet to reduce dyslipidemia and to protect against lipid peroxidation and inflammation.	2C
Counselling	**CARI (2013) [15]**	
	We suggest that patients with progressive CKD have individualised dietary interventions involving an appropriately qualified dietitian.	
	**NICE (2008) [13]**	
	Where the clinician in discussion with the patient has decided that dietary intervention to influence progression of CKD is indicated, an appropriately trained professional should discuss the risks and benefits of dietary protein restriction, with particular reference to slowing down the progression of disease *vs.* protein-calorie malnutrition.	2C
	Where dietary intervention is agreed this should occur within the context of education, detailed dietary assessment and supervision to ensure malnutrition is prevented.	Not graded
	Offer dietary advice to people with progressive CKD concerning potassium, phosphate, protein, calorie and salt intake when indicated.	
Conservative management	**CMA (2008) [11]**	
	Renal programs and care providers for patients with progressive chronic kidney disease who choose not to pursue renal replacement therapies should ensure patients have access to an interdisciplinary team to provide comprehensive conservative management. All chronic kidney disease programs and care providers should have a mechanism by which to develop documents and processes for advanced-care planning. Comprehensive conservative management protocols should include symptom management, psychological care and spiritual care. Coordinated end-of-life care should be available to patients and families.	Not graded

The Australian CARI guidelines, shown in Table 3, state that a protein-controlled diet consisting of 0.75–1.0 g/kg/day, is recommended for adults pre-dialysis (Stages 3–4) [15]. The administration of a low protein diet (<0.6 g/kg/day) to slow renal failure progression is not justified when the reported clinically modest benefit on glomerular filtration rate decline is weighed against the concomitant significant declines in clinical and biochemical parameters of nutrition [15]. It is the most recent of the international guidelines assessing this question and is at odds with the systematic reviews [22,23].

The British Dietetic Association’s guidelines on protein intake in both haemodialysis (HD) and peritoneal dialysis (PD) recommend a lower level of protein intake than previous guidelines at 1.1 g/kg ideal body weight/day for those undergoing maintenance haemodialysis and 1.0–1.2 g/kg ideal body weight/day for those on maintenance peritoneal dialysis [19]. These recommendations are graded C using the Scottish Intercollegiate Guideline Network criteria, that is based on well-conducted cohort or case control studies with a low risk of confounding and a moderate probability that the relationship is causal [12]. The authors emphasise the importance of adequate energy (126–167 kJ/day in HD and 146 kJ/day for PD in adults under 60 years and 126–146 kJ/day for those over 60 years). This recommendation is slightly lower than previously recommended and is based on medically well patients with stable body weights and the authors caution when applying these recommendations to less well patients [19].

The guidelines on vitamin D (Table 3) focus on the general population decline in serum 25 hydroxy vitamin D and methods to address this in early CKD (Stages 1–4) [15]. In later stages of disease, recent guidelines focus on the combined effects of calcium, phosphate, parathyroid hormone (PTH) and vitamin D on outcome [14,17]. The cohort study by Wang *et al.* aimed to explore the relationship between serum 25(OH)-hydroxy vitamin D (25(OH)D) in PD patients and long term clinical outcomes [36]. They found that 87% of the cohort were deficient or insufficient in 25(OH)D (*i.e.*, <75 nmol/L) and that lower serum 25(OH)D levels were associated with an increased risk of cardiovascular events but not long term mortality [36]. The effects of oral paricalcitol supplementation on biochemical markers (including proteinuria) have been studied in both pre-dialysis and early CKD patients (Stages 1–4). A small, six month randomized controlled trial (RCT) found a modest effect size of oral paricalcitol supplementation of 1 μg/day *vs*. placebo, with the intervention group demonstrating a 17.6% decrease in spot urinary protein-creatinine ratio *vs.* a 2.9% increase for controls (*p* = 0.04) [34]. It was also noted in this study that serum iPTH fell significantly amongst those who received paricalcitol supplementation (*p* = 0.01) [34]. Agarwal *et al.* similarly found that oral paricalcitol supplementation (mean dose 9.5 μg/week) was significantly associated with 51% *vs.* 25% (*p* = 0.004) reduction in proteinuria in the intervention group compared to controls and 3.2 greater odds for a reduction in proteinuria independent of treatment for Renal Angiotensin Aldosterone blockade [35]. The KDIGO guidelines (see Table 3) recommend calcitriol or other vitamin therapy in those with elevated parathryroid hormone [17]. The CARI guidelines while recommending vitamin D therapy in early kidney disease for those with elevated PTH warn against the risk of vitamin D therapy in the face of elevated serum calcium and phosphate levels, which should be monitored regularly [15].

The evidence for the modification of fat in CKD to moderate cardiovascular outcomes is limited. Beavers *et al.* found that supplementation of 6 g omega-3 fatty acids had no effect on total homocysteine levels in HD patients over 6 months [37]. Saltissi *et al.* found that dietary compliance was a major issue [38]. A dialysis dietary prescription modified to meet the National Heart Foundation guidelines of reduced intake of saturated fat and cholesterol, led to a significant reduction in total cholesterol and low density lipoprotein (LDL) cholesterol in HD patients with little effect in PD patients [38]. All guidelines published since 2006, recommend controlling salt intake below 100 mmol sodium/day (2.3 g sodium) as an important feature of managing hypertension [11,12,15,18], although not addressed at any particular stage of CKD. One randomized controlled double blind crossover study of 34 patients with proteinuria and without diabetes was located reporting the effect of a low sodium diet (50 mmol/day) being as efficacious as treatment with hydrocholorothiazide (an angiotensin receptor II antagonist) at reducing proteinuria and blood pressure when combined with a diuretic [39]. Sodium restriction itself exerted a modest, yet significant, antiproteinuric effect [37]. Actively restricting sodium to less than 100 mmol/day (5 g salt) in those undergoing haemodialysis resulted in less hypertensive medications used (7% *vs*. 42%), better ventricular function and less intradialytic hypotension compared to those whose blood pressure was controlled by medication [40]. Using sodium excretion as a surrogate for sodium intake, Boudville showed that excretions in the lowest tertile (114 mmol/day) resulted in significantly fewer hypertensive medications (2 *vs*. 2.7, *p* = 0.01) used in those with GFR < 30 mL/min, than those in the highest tertile (166.7 mmol/day). This effect was even more marked in those with GFR ≤ 15 mL/min [41].

The effect of dietary fibre supplements and a high fibre diet, on patient reported symptoms of constipation amongst a PD population, suggested that 6–12 g/day of partially hydrolysed guar gum added to usual intake was as effective as usual laxative treatment for preventing constipation in the majority of included PD patients and was associated with less unfavorable side effects [42]. Both Saltissi and Sutton studies were case series without control groups [38,42].

Compliance with diet prescription remains an issue. Twenty-eight per cent (28%) of participants under-reported protein intake in both a very low protein diet (VLPD) of 0.3 g/kg/day plus keto-acids (KA) diet to a level of 0.66 g/kg/day and a low protein diet (LPD) of 0.6 g/kg/day in pre-dialysis patients. While compliance was poor in both groups, the prescription of the VLPD + KA delivered improved biochemical markers, with significant improvements noted in serum urea nitrogen, serum bicarbonate and urinary phosphorous [31]. In 423 pre-dialysis patients (Stages 4 and 5) randomized to receive two different protein levels, LPD (0.55 g/kg/day) or a Moderate Protein Diet (MPD) of 0.8 g/kg/day, for 3 months with follow up to 48 months there were no differences between groups at 6 and 18 months, however there was greater compliance with the MPD [32]. In a case control study of Stage 3–5 CKD patients, Kanazawa demonstrated the compliant group, with dietary protein intakes maintained at 0.69 g/kg body weight/day, had smaller decline in GFR, however no measures of change in body composition were recorded and there was no difference in health-related quality of life [33].

In an open RCT in 28 PD patients, randomized to receive a powdered egg-albumin protein supplement (30 g/day providing 22 g protein) *vs*. nutrition counselling over six months resulted in significantly improved serum albumin (2.64 ± 0.35 *vs.* 3.05 ± 0.72 g/dL), energy intake (1331 ± 342 *vs.* 1872 ± 698 kcal/day), protein intake (1.0 ± 0.3 *vs.* 1.7 ± 0.7 g/kg) and nPNA (1.00 ± 0.23 *vs.* 1.18 ± 0.35 g/kg/day) amongst the intervention group when compared to baseline measures, and frequency of moderate-severe malnutrition decreased 28% in the intervention *vs*. 6% in the control group [45]. Interestingly, compliance in this study was reported as 90%.

Teixido-Planas *et al.* conducted a 12 month open RCT of 65 PD participants, comparing daily consumption of 200 mL 1.0 kcal/mL liquid oral nutrition support (ONS), in addition to usual dietary intake, against those who consumed only their usual dietary intake [43]. Based on an “intention to treat” analysis, only an improvement in total lymphocyte count (*p* = 0.0066) between intervention and controls reached significance. The supplement was not found to be suitable for long term use due to non-compliance with 31% of the intervention group dropping out. A similar study by Caglar *et al*. with ONS showed improvements in albumin, pre-albumin and SGA [44], however the compliance rate (32%) was similar, with a 46% dropout rate.

Five studies investigating the effect of structured dietetic counseling on compliance with dietary prescriptions have shown differing results. Campbell *et al.* randomized 56 pre-dialysis patients (Stages 4 and 5) to fortnightly, individualized counseling on a prescription of 0.75 g/kg/day protein and 145 kJ/kg/day energy *vs*. written education material for 3 months [46]. The intervention group had a significantly lower reduction in body cell mass and improvement of 17.7 kJ/kg/day energy intake and subjective global assessment (SGA). Improvements in nutritional status in the intervention group translated to significant improvements in the symptoms, cognitive functioning and vitality subscales in the Kidney Disease Quality of Life tool, KDQoL [47]. Sullivan also showed in 279 HD patients in a cluster RCT for 3 months that counseling on reducing phosphates in foods compared to usual care significantly reduced serum phosphate levels by 0.6 mg/dL, largely through improvements in food label reading [48]. Conversely, Morey in 67 HD patients randomized to monthly *vs*. 6 monthly counseling was unable to maintain a reduction in serum phosphate of 0.25 mg/dL at 3 months, at the 6 month follow-up [49]. A retrospective cohort study over 2 years of 65 HD patients receiving a 6 monthly dietetic review with intensive follow-up for nutrition parameters falling below recommended levels, showed a significant reduction in malnutrition (SGA-B reducing from 14% to 3%), maintained serum albumin, potassium an dry weight and significant reduction in serum phosphate [50].

The evidence for the effect of nutrients in peritoneal dialysis solution is limited to two small studies [51,52]. Improvements in overall protein balance improved in a randomized cross over study of 8 patients over 14 days [51] and also in another study which was open labeled and not controlled in 16 patients over 3 months [52]. The use of intradialytic parenteral nutrition (IDPN) solution in HD patients has also only been conducted in small studies, showing improvements in hepatic albumin synthesis and whole body fat free mass [53,54,55,56,57]. In the cross over study of 8 patients using both IDPN and oral supplements, the oral administration resulted in persistent anabolic benefits in the post dialysis phase, which was not seen with IDPN [55]. In a case series over 12 months of 24 malnourished PD patients, in which there was significant attrition >50%, the IDPN was associated with increased body weight and improved serum albumin levels [57]. A small case series in haemodialysis patients, using Percutaneous Endoscopic Gastrostomy feeding showed improvements in anthropometric measures over a 3 month period [58].

## 4. Discussion

The focus of guidelines on nutrition and CKD published since 2006 has been on early prevention and lifestyle modification required to prevent progression to ESRD [11,12,15] or the management of renal bone disease [17]. The KDOQI guidelines on nutrition have not been updated since 2000 [59]. Addressing general population’s sub-optimal serum vitamin D levels, as well as in early CKD is a priority. Other chronic diseases, such as obesity, diabetes and hypertension, which affect the population at large, require management to prevent progression to CKD [15]. The treatment of these diseases has a large nutrition component which needs to be recognized [6].

The approach for managing elevated serum phosphate, through the use of phosphate binders as an adjunct to the restriction of dietary intake, has also been recognized. The KDIGO guidelines continue to recommend restricting dietary phosphate in combination with other treatments, however the evidence is poor [5]. The CARI guidelines state clearly that restriction of diet runs the risk of precipitating malnutrition and thus has promoted moderate restrictions in protein, phosphate and sodium in the pre-dialysis period to levels commensurate with the general population [15]. The studies, specifically looking at dietitian led control of phosphate intake, showed promise but further studies need to be conducted on the frequency of dietetic counseling to ensure long term impact on dietary control [49,50].

Issues of compliance with restricted diets remains a weakness in many of the studies reported here and those promoting regular contact with a dietitian mostly report improved outcomes. The previous DAA guidelines [7] have had wide currency with dietitians in Australia and New Zealand and are largely still relevant in the present day. These guidelines used the Nutrition Care Process (NCP) to guide the development of clinical questions. The NCP consists of nutrition assessment, diagnosis, intervention and monitoring and evaluation [8] and is outcome driven in that nutritional parameters collected as part of the nutrition assessment and addressed through the nutrition prescription, are then re-assessed or evaluated to establish the impact of the nutrition intervention. These outcomes commonly include intermediate outcomes, such as nutrient intake, anthropometric measures and biochemical markers. Studies on the effect of nutrition prescription on clinical outcomes, such as mortality, hospitalization or cost are limited. While the NCP is useful for practical purposes, grading of evidence in line with international recommendations on harmonizing guidelines is still required [21]. One advantage of these nutrition guidelines is the rigorous independent review process undertaken using the Appraisal of Guidelines for Research and Evaluation (AGREE) tool, which has been recommended for future evaluation of guidelines [60]. Areas requiring most revision for the future include recommendations on vitamin D and phosphate. Further studies on the effect of intradialytic parenteral nutrition and enteral support on dialysis are also warranted.

## 5. Conclusions

Overall, the body of evidence supporting nutritional interventions for improving patient outcomes in CKD is primarily based on low level evidence or isolated randomized clinical trials. Much of the evidence around dietary prescription relies on retrospective and uncontrolled cohort studies and the quality of the body of evidence is poor. Most outcomes assessed are generally biochemical endpoints, such as change in serum levels, rather than clinical ones, such as mortality, hospitalization, cost and patient quality of life. There is general agreement across guideline recommendations for the levels of protein in early CKD and on dialysis; however, guidance on the use of very low protein diets with keto-analogues in conservative treatment of those with GFR < 15 mL/min is warranted. While the evidence from a few observational trials suggests that these diets pose no greater risk on mortality than dialysis as treatment, better controlled trials are required to confirm this. Further research on the optimal intakes of sodium, phosphate, fats and fibre in well controlled studies are required, as are studies into micronutrients and other components such as antioxidants. Studies on sun exposure combined with diet are required to determine optimal vitamin D status. The collaborative effort to use a global approach to international guidance in management of chronic kidney disease is welcome. While more evidence based studies are warranted, the customary nutrition prescription remains satisfactory with the exception of Vitamin D and phosphate. In these two areas, additional research is urgently needed, given the potential of adverse outcomes for the CKD patient. The role of nutrition in the management of CKD is important and needs to be included in further promotion of research outcomes and future guidelines.

## Appendix

**Appendix 1:** Grading of evidence for different guidelines.

Grading Body	Best evidence (A/1A/Strong)		Good Evidence (B/Fair)		Mixed Evidence (C)		Weak Evidence (D)	
	**A—Excellent**		**B****—Good**		**C****—Satisfactory**		**D****—Poor**	
NHMRC. National Health and Medical Research Council, Australia (2009) [20]	Body of evidence can be trusted to guide practice. Several level I or II studies with low risk of bias; Excellent consistency across studies; Very large clinical impact; Results are directly generalisable to target population; Results are directly applicable to the Australian healthcare context.		Body of evidence can be trusted to guide practice in most situations. One or two level II studies with low risk of bias or systematic review of multiple level III studies with low risk of bias. Most studies are consistent and inconsistencies can be explained. Substantial clinical impact; Results are directly generalisable to target population with some caveats; Results are directly applicable to the Australian healthcare context with few caveats.		Body of evidence provides some support for recommendation(s) but care should be taken in its application. Satisfactory level III studies with low risk of bias or level I or II studies with moderate risk of bias. Some inconsistency reflecting genuine uncertainty around question. Moderate clinical impact; Not directly generalisable to target population but could be sensibly applied. Results are probably applicable to the Australian healthcare context with some caveats.		Body of evidence is weak and recommendation must be applied with caution. Level IV studies or level I to III studies with high risk of bias. Evidence is inconsistent; Slight or restricted clinical impact. Not directly generalisable to target population hard to judge whether it is sensible to apply. Results are not applicable to the Australian healthcare context.	
SIGN Scottish Intercol-legiate Guidelines Network 2008 [12]	**A**		**B**		**C**		**D**	**Good Practice Points**
	At least one meta-analysis, systematic review, or RCT rated as 1++, and directly applicable to the target population; OA body of evidence consisting principally of studies rated as 1+, directly applicable to the target population, and demonstrating overall consistency of results.		A body of evidence including studies rated as 2++, directly applicable to the target population, and demonstrating overall consistency of results; OR; Extrapolated evidence from studies rated as 1++ or 1+.		A body of evidence including studies rated as 2+, directly applicable to the target population, and demonstrating overall consistency of results; OR; Extrapolated evidence from studies rated as 2++.		Evidence level 3 or 4; Extrapolated evidence from studies rated as 2+.	Recommended best practice based on the clinical experience of the guidelines development group.
Canadian Society Nephrology (2008) [11]	High quality RCT or meta-analyses with adequate power and clinically important outcomes.		High quality RCT or meta-analyses with adequate power but outcome is a validated surrogate or results need to be extrapolated from study population to real population OR; High quality RCT or meta-analyses with inadequate power but with clinically important or validated surrogate outcome		High quality RCT or meta-analyses with adequate power but outcome is neither clinically important or a validated surrogate outcome OR; Observational study with statistically significant results and outcome is clinically important or a validated surrogate AND study population is representative of population recommendation is for OR results can be extrapolated from study population to real population.		High quality RCT or meta-analyses with inadequate power and neither clinically important nor validated surrogate outcomes OR; Observational study with statistically significant results but neither clinically important nor validated surrogate outcome OR; Observational study with inadequate power and applicability of the study is irrelevant.	
KDIGOKidney Disease Improving Global Outcomes (2013) [5]	**A High**		**B Moderate**		**C Low**		**D Very Low**	
	We are confident that the true effect lies close to that of the estimate of the effect. Level 1 “We recommend”. Most peoplein situation would want the recommended course of action and only a small proportion would not. The recommendation can be evaluated as a candidate for developing a policy or a performance measure.		The true effect is likely to be close to the estimate of the effect, but there is a possibility that it is substantially different. Level 2 “We suggest”; The majority of people *in situ* ation would want the recommended course of action, but many would not. The recommendation is likely to require substantial debate and involvement of stakeholders before policy can be determined.		The true effect may be substantially different from the estimate of the effect.		The estimate of effect is very uncertain, and often will be far from the truth.	
	**A**		**B**				**C**	
KDOQI. National Kidney Foundation*—*Kidney Disease Outcome Quality Initiative (2002) [61]	It is strongly recommended that clinicians routinely follow the guidelines for eligible patients. There is strong evidence that the practice improves health outcomes.		It is recommended that clinicians routinely follow the guideline for eligible patients. There is moderately strong evidence that the practice improves health outcomes				It is recommended that clinicians consider following the clinical practice recommendation for eligible patients. This recommendation is based on either weak evidence or on the opinions of the work group and reviewers that the practice might improve health outcomes.	
	**Strong**				**Fair**		**Weak**	**Consensus**
ADA. American Dietetic Association (2010) [18]	The workgroup believes the benefits of the recommended approach clearly exceed the harms (or that harms clearly exceed benefits in the case of a strong negative recommendation) and that the quality of the supporting evidence is excellent/good (grad I or II).				The workgroup believes the benefits exceed the harms (or that harms clearly exceed benefits in the case of a strong negative recommendation) but the quality of evidence is not as strong (grade II or III)		Quality of evidence that exists is suspect or well done studies (grade I, II or III) show little clear advantage to one approach *versus* another. Patient preferences should have a substantial influencing role in patient care.	A consensus recommendation means that expert opinion (grade IV) supports the guideline recommendation even though the available scientific evidence did not present consistent results, or controlled trials were lacking.
CARI Caring for Australians with Renal Impairment (2013) [15]	**1A**	**2A**	**1B**	**2B**	**1C**	**1D**	**1D**	**2D**
	Strong recommendation High quality evidence. Consistent evidence from well performed RCTs or overwhelming evidence of some other form. Further research is unlikely to change our confidence in the estimate of benefit and risk. Strong recommendations can apply to most patients in most circumstances without reservation.	Weak recommendation High quality evidence. Consistent evidence from well performed RCTs or overwhelming evidence of some other form. Further research is unlikely to change our confidence in the estimate of benefit and risk. Clinicians should follow a strong recommendation unless there is a clear rationale for an alternative approach.	Strong recommendation. Moderate quality evidence. Evidence from RCTs with important limitations (inconsistent results, methods flaws, indirect or imprecise), or very strong evidence of some other research design. Further research may impact on our confidence in the estimate of benefit and risk. Strong recommendation and applies to most patients.	Weak recommendation. Moderate quality evidence. Evidence from RCTs with important limitations (inconsistent results, methods flaws, indirect or imprecise), or strong evidence of some other research design. Further research may change the estimate of benefit and risk. Clinicians should follow a strong recommendation unless a clear and compelling rationale for an alternative approach is present.	Strong recommendation. Low quality evidence. Evidence from observational studies, unsystematic clinical experience, or from RCTs with serious flaws. Any estimate of effect is uncertain. Strong recommendation, and applies to most patients. Some of the evidence base supporting the recommendation is, however, of low quality.	Weak recommendation. Low quality evidence. Evidence from observational studies, unsystematic clinical experience, or from RCTs with serious flaws. Any estimate of effect is uncertain.	Strong recommendation. Very low quality evidence; Evidence limited to case studies. Strong recommendation based mainly on case studies and expert judgement.	Weak recommendation. Very low quality evidenceEvidence limited to case studies and expert judgementVery weak recommendation, other alternatives may be equally reasonable.
Grading of Recommendations Assessment Development Evaluation (GRADE) [59]	**High**		**Moderate**	**Low**			**Very low**	
	We are very confident that the true effect lies close to that of the estimate of the effect. Further research is very unlikely to change our confidence in the estimate of effect.		We are moderately confident in the effect estimate: The true effect is likely to be close to the estimate of the effect, but there is a possibility that it is substantially different. Further research is likely to have an important impact on our confidence in the estimate of effect and may change the estimate.	Our confidence in the effect estimate is limited: The true effect may be substantially different from the estimate of the effect. Further research is very likely to have an important impact on our confidence in the estimate of effect and is likely to change the estimate.			We have very little confidence in the effect estimate: The true effect is likely to be substantially different from the estimate of effect. Any estimate of effect is very uncertain.	
